# Preliminary examination of reliability and validity of the Japanese child anxiety impact scale-parent version in Japanese community sample

**DOI:** 10.1007/s12144-021-02437-5

**Published:** 2021-10-31

**Authors:** Sho Okawa, Honami Arai, Hideki Nakamura, Yuko Urao, Tessa Reardon, Sophie Giles, Eiji Shimizu

**Affiliations:** 1 Faculty of Humanities, Wayo Women’s University, 2-3-1, Konodai, Ichikawa-shi, Chiba, 272-8533 Japan; 2 Research Center for Child Mental Development, Chiba University, 1-8-1, Inohana, Chuo-ku, Chiba-shi, Chiba, 260-8670 Japan; 3 Department of Cognitive Behavioral Physiology, Chiba University, 1-8-1, Inohana, Chuo-ku, Chiba-shi, Chiba, 260-8670 Japan; 4 Center for Research on Counseling and Support Services, Tokyo University, 7-3-1 Hongo, Bunkyo-ku, Tokyo 113-0033 Japan; 5 Department of Experimental Psychology, University of Oxford, Radcliffe Observatory Quarter, Anna Watts Building, Woodstock Road, Oxford, OX2 6GG UK; 6 Department of Psychiatry, University of Oxford, Warneford Hospital, Oxford, OX3 7JX UK

**Keywords:** Childhood anxiety, Child anxiety impact scale-parent version, Japanese children, Japan, Validity, Reliability

## Abstract

The child anxiety impact scale-parent version (CAIS-P) is a useful measure to assess the impact of anxiety on a child’s daily life; however, a Japanese version of the CAIS-P has not been developed, and whether the CAIS-P can be utilized in Eastern countries remains unascertained. The purpose of this study was to develop a Japanese version of the CAIS-P and examine its reliability and validity. Parents of 400 children (aged 7 to 15 years) from the Japanese community completed the CAIS-P. A confirmatory factor analysis indicated that the factor structure of the original CAIS-P, consisting of school activity, social activity, and home/family activity factors, provided a good fit for the Japanese version of the CAIS-P. Estimated Spearman’s correlation coefficients showed moderate correlations between the total and factor scores of the CAIS-P, anxiety symptoms (Spence Child Anxiety Scale-parent version), and depressive symptoms (Child Depression Inventory). Furthermore, the item response theory model revealed that each factor of the CAIS-P is a high information reliable measure for children with high trait anxiety. These results provide support for the Japanese version of the CAIS-P’s factorial validity, convergent validity, and reliability and its potential for application in child anxiety research in Japan.

Anxiety disorders in childhood are the most prevalent mental health disorders associated with impairment in everyday life (Cartwright-Hatton et al., 2006). Childhood anxiety disorders are often comorbid with depression, leading to more severe symptoms among those who have both compared with those who have only one of the two disorders (Melton et al., 2016). Half of the anxiety disorders across a lifespan first appear by 11 years of age and can lead to other problems, such as other anxiety disorders, mood disorders, and substance abuse (Bittner et al., 2007; Compton et al., 2002; Kessler et al., 2005). Cognitive behavior therapy (CBT) is an effective treatment for childhood anxiety with a remission rate of 49.4% (James et al., 2020). For assessment of anxiety disorders in children, the Anxiety Disorders Interview Schedule for Diagnostic and Statistical Manual of Mental Disorders-IV (DSM-IV) for Children-Child and Parent Version (ADIS-IV-C/P) is considered the gold standard (Silverman & Albano, 1996). Indeed, the ADIS-IV-C/P is often used to determine eligibility and assess outcomes in clinical trials of treatment for child anxiety disorders. However, it can be cumbersome for clinicians, children, and their parents as it needs to be conducted by trained clinicians and requires an average of 133 min to administer (Lyneham & Rapee, 2005). Therefore, questionnaire measures are frequently used to assess child anxiety in clinical, research, and community settings (Creswell et al., 2020).

Child anxiety questionnaire measures typically assess the presence and severity of a child’s anxiety symptoms. One of the most widely used child- and parent-report questionnaire measures to assess a child’s anxiety symptoms is the Spence Child Anxiety Scale (SCAS-C/P; Spence, 1998). The SCAS-C/P comprises 38 items designed to assess symptoms of social phobia, panic/agoraphobia, generalized anxiety, obsessive–compulsive behaviors, separation anxiety, and specific phobias. The psychometric properties of the SCAS-C/P are well established in both clinical and community populations (e.g., Brown-Jacobsen et al., 2011; Spence, 1998), and the questionnaire has been translated into over 20 languages, including Japanese (Ishikawa et al., 2012; Ishikawa et al., 2019; Ramme, 2018). The Multidimensional Anxiety Scale for Children (MASC) is also translated into Japanese and used in child anxiety research (Ando, 2008). However, although the reliability and validity of the Japanese version of MASC is confirmed in community samples (Ando, 2008), its psychometric properties have not been evaluated among children with anxiety disorders. In addition, the parent report version of MASC has not been translated in Japanese, and only the child report version is used in Japan. Consequently, child anxiety studies among clinical populations in Japan tend to use the SCAS-C/P. However, anxiety disorder diagnoses are characterized by the presence of *both* anxiety symptoms *and* functional interference, and relying exclusively on the SCAS-C/P may fail to capture the extent to which a child’s anxiety symptoms interfere with daily life. Indeed, recent guidance on reporting outcomes in child anxiety treatment trials recommends including a measure of functional interference in the trials (Creswell et al., 2020

A small number of questionnaire measures designed to assess interference associated with child anxiety symptoms are available in English, including the Child Anxiety Impact Scale (CAIS-C/P). The CAIS-C/P is a child and parent report questionnaire, comprising three subscales to assess interference related to school activities, social activities, and home/family activities; it has good internal consistency and construct validity (Langley et al., 2014). Moreover, Evans et al. (2016) found that the CAIS-P was a good predictor of recovery from anxiety disorder diagnoses, and it was more successful at identifying recovery from anxiety disorders than the SCAS-C/P. However, currently there are no questionnaire measures designed to assess functional interference associated with child anxiety symptoms that are available in Japanese. As a result, child anxiety assessments in clinical trials and other settings in Japan are highly dependent on the SCAS-C/P (e.g., Ishikawa et al., 2019).

Using consistent child anxiety measures internationally facilitates comparisons of treatment outcomes across contexts (Creswell et al., 2021) and enables exploration of cultural differences in the expression and interpretation of child anxiety disorders (Ishikawa et al., 2014). Indeed, some anxiety-related behaviors that are perceived as maladaptive in Western countries are often interpreted differently in Eastern countries. For example, in Eastern countries like Japan and China, which emphasize harmony within social groups, fear and socially withdrawn behaviors are often perceived as adaptive behaviors (Heinrichs et al., 2006; Kleinknecht et al., 1997); they have been found to be associated with peer acceptance and positive school behavior among Chinese children (Chen et al., 2009) and suppression of anti-social behavior in Japanese boys (Usukura & Hamaguchi, 2015). These findings indicate that the impact of child anxiety symptoms on everyday life might differ across Western and Eastern cultural contexts; however, in the absence of a suitable measure that can be used across countries and cultures these potential differences remain unexplored.

The purpose of the present study was to develop a Japanese version of the CAIS-P and examine its reliability and factorial, convergent, and divergent validity in a community sample. We set out to provide preliminary evidence for the suitability of the CAIS-P for use in Japan and its potential for future application in clinical settings. We plan to use findings from this initial evaluation to inform the future development of a child-reported Japanese version of the CAIS. The availability of a reliable and valid Japanese version of the CAIS would enable child anxiety assessments in Japanese settings to incorporate a questionnaire measure of the impact of child’s anxiety symptoms.

First, we hypothesized that the factor structure of the original CAIS-P (Langley et al., 2004) is supported in the Japanese version of the CAIS-P. Second, we hypothesized that the Japanese version of the CAIS-P moderately correlates with SCAS-P. Third, as the CAIS-P measures the impact of children’s anxiety on daily functioning, we expected to find a stronger correlation between the CAIS-P and SCAS-P than between the CAIS-P and depressive symptoms measured by the Children’s Depression Inventory (CDI). In addition, we used the item response theory (IRT) approach to examine the reliability and amount of information on each factor in the CAIS-P. A major advantage of IRT is that this approach is independent of the respondents’ characteristics (Bortolotti et al., 2012). IRT calculates latent traits of respondents, but the parameters derived from IRT do not depend on the respondents’ characteristics. Since the results of IRT can be generalized to a different population, the findings provide preliminary evidence for the CAIS-P's potential utility in Japan.

## Methods

### Participants

Parents of 400 children (aged 7 to 15 years) were recruited online via Rakuten Insight to participate in this study. Rakuten Insight is a research company that conducts surveys of Rakuten service registrants. Rakuten Insight maintains data quality by monitoring unauthorized registration of respondents at the stated period. All the participants received points that they could redeem with Rakuten services. Each parent completed questionnaire measures for one child at one time point via an online platform. The inclusion criteria required that parents and their children be aged between 18 and 65 years, and 7 and 15 years, respectively. The parents’ and their children’s characteristics are illustrated in Table 1. All the participants are Japanese and currently live in Japan. Ethical approval was obtained from the Ethics Committee of Chiba University (study number 3924). Informed consent was obtained from all the participants. This study was conducted in accordance with the Declaration of Helsinki. All the data were collected in December 2020.

**Table 1:** Demographic data

Characteristics		Child
Age: Mean (SD)		11.34 (2.52)
Gender: n (%)	Male	230 (57.50)
	Female	170 (42.50)
School year: n (%)	1	21 (5.25)
	2	39 (9.75)
	3	40 (10.00)
	4	37 (9.25)
	5	50 (12.50)
	6	44 (11.00)
	7	53 (13.25)
	8	56 (14.00)
	9	55 (13.75)
	10	5 (1.25)
		Parent
Age: Mean (SD)		45.67 (5.92)
Gender: n (%)	Male	275 (68.75)
	Female	125 (31.25)
Marital status: n (%)	Married	376 (94.00)
	Single	15 (3.75)
	Other	9 (2.25)
Relationship: n (%)	Father	274 (68.50)
	Mother	125 (31.25)
	Grandparent	1 (0.00)

## Measures

### Spence Child Anxiety Scale-Parent version (SCAS-P; Nauta et al., 2004)

The SCAS-P is a 38-item parent-report measure with items rated on a 4-point Likert-type scale ranging from 0 (*never*) to 3 (*always*). The SCAS-P is used to assess symptoms of anxiety disorders in children. The total of all 38 items reflects the level of anxiety symptoms in each child, with higher scores reflecting higher levels of anxiety symptoms. The Japanese version of the SCAS-P demonstrates adequate internal consistency (α = 0.88), and is moderately correlated (*r* = 0.51) with anxiety/depression scores from the Child Behavior Checklist, which represents good convergent validity (Ishikawa et al., 2014). In the current sample, the internal consistency was also good (α = 0.96).

### Child Depression Inventory (CDI; Kovacs, 1985)

The CDI is a 27-item measure to assess a child’s depressive symptoms. Each item in the CDI has three descriptions for depressive symptoms, with responses scored from 0 to 2. Thirteen items are reverse-scored; higher scores reflect higher levels of depressive symptoms in children. The Japanese version of the CDI shows good internal consistency (α = 0.83), and is moderately correlated with depression items from the Youth Self Report (*r* = 0.63), supporting its convergent validity (Mashida et al., 2009). The internal consistency was also good in the current sample (α = 0.91). In this study, we instructed parents to report their child’s depressive symptoms.

### Child Anxiety Impact Scale-Parent version (CAIS-P; Langley et al., 2004)

The CAIS-P is a 27-item parent self-report measure to assess the impact of a child’s anxiety on three categories: school activities, social activities, and home/family activities. The measure shows good internal consistency and convergent validity (Langley et al., 2014). In this study, each item was rated on a 4-point Likert scale ranging from 0 *(not at all*) to 3 (*very much*). The total score of all items in each category indicated the level of impact of a child’s anxiety on each category. We also summed all 27 items to see the overall impact of anxiety on children’s daily lives.

We developed the Japanese version of the CAIS-P by using the translation and back-translation procedure according to the COSMIN checklist (Mokkink et al., 2012). Prior to developing the Japanese version of the scale, we obtained permission from the original author, and the first author who translated the English version of CAIS-P into Japanese. Before commencing the back-translation process, the first author discussed the clarity and language expression of the translation with a psychiatrist, a clinical psychologist, and two nurses, and modified the translation of instructions and items accordingly. Subsequently, a translator from an agency translated the preliminary Japanese version of the CAIS-P back to English. Two researchers with expertise in child anxiety disorders and experience in using the CAIS-P confirmed the appropriateness and replicability of the back-translation from the original CAIS-P. The wording of the instructions and two items were amended to reflect the original CAIS-P’s intended meaning in the final version of the CAIS-P in Japanese.

## Statistical Analysis

All analyses were conducted using Stata 16 software, except for confirmatory factor analysis and IRT, which were conducted using Mplus version 8. Owing to the skewed distribution of CAIS-P (skewness = 2.84) and inability to assume normal distribution, we used non-parametric estimation for the following analyses. To examine the factorial validity, we conducted confirmatory factor analyses (CFA) using a weighted least squares with means and variance adjustment (WLSMV) method of estimation. We evaluated the model fit using the comparative fit index (CFI), root mean square error of approximation (RMSEA), and standardized root mean squared residual (SRMR). The following values were considered as acceptable cut-off values for each fit index: (1) CFI > 0.95, (2) RMSEA < 0.08, and (3) SRMR < 0.08 (Browne & Cudeck, 1993; Hu & Bentler, 1999). After confirming the factor structure, we estimated Spearman’s rank correlation coefficient to examine the convergent and discriminant validity of the Japanese version of the CAIS-P. As the distribution of the CAIS-P was skewed, we were unable to use a parametric test so Spearman’s rank correlation was used instead of the Pearson correlation. To test whether the correlation between CAIS-P and SCAS-P was larger than the correlation between CAIS-P and CDI, we used the CORTESI procedure in Stata (Caci, 2000). We also estimated that the partial correlation coefficient controlled the effect of depressive symptoms. The correlation coefficient 0.10 is considered weak, 0.30 is considered medium, and 0.50 is considered large (Cohen, 1988). Furthermore, to examine the reliability and the amount of information in each factor in CAIS-P, we conducted a graded response model of IRT with the WLSMV estimator. Since IRT is only applicable to unidimensional measures, we confirmed the unidimensionality of each factor using principal component analysis prior to IRT. We estimated the discrimination and difficulty parameters for each item, and the test information function for each factor. A higher discrimination parameter indicated the item’s ability to differentiate a child who was highly impacted by anxiety from those who were not. The discrimination parameter between 0.01-0.34 is very low, between 0.35-0.64 is low, between 0.65–1.34 is moderate, between 1.35–1.69 is high, and above 1.70 is very high (Baker, 2001). The difficulty parameter indicates the trait level of respondents; 50 percent of the population answered as belonging to category m or higher. The difficulty parameter around -2 is very low, around 0 is moderate, and around 2 is very high (Hambleton et al., 1991). The sample size for the current study was set according to the recommended sample size for confirmatory factor analysis and IRT, which was 300 and 375, respectively (De Alaya, 1994; Tabachnick & Fidell, 2007).

## Results

Table 2 shows the mean and standard deviation of each measure.

**Table 2:** Mean and standard deviation of each measure (*n* = 400)

	Mean	SD
SCAS-P	15.07	17.50
CDI	9.41	7.91
CAIS-P-Total	6.87	11.02
School	3.13	4.64
Social	2.47	4.71
Home/Family	1.27	2.72

CAIS-P = Child Anxiety Impact Scale-Parent version, School = School activity, Social = Social activity, Home/Family = Home/Family activity, SCAS-P = Spence Child Anxiety Scale-Parent version, CDI = Child Depression Inventory

### Factorial Validity of CAIS-P

**Fig. 1:**
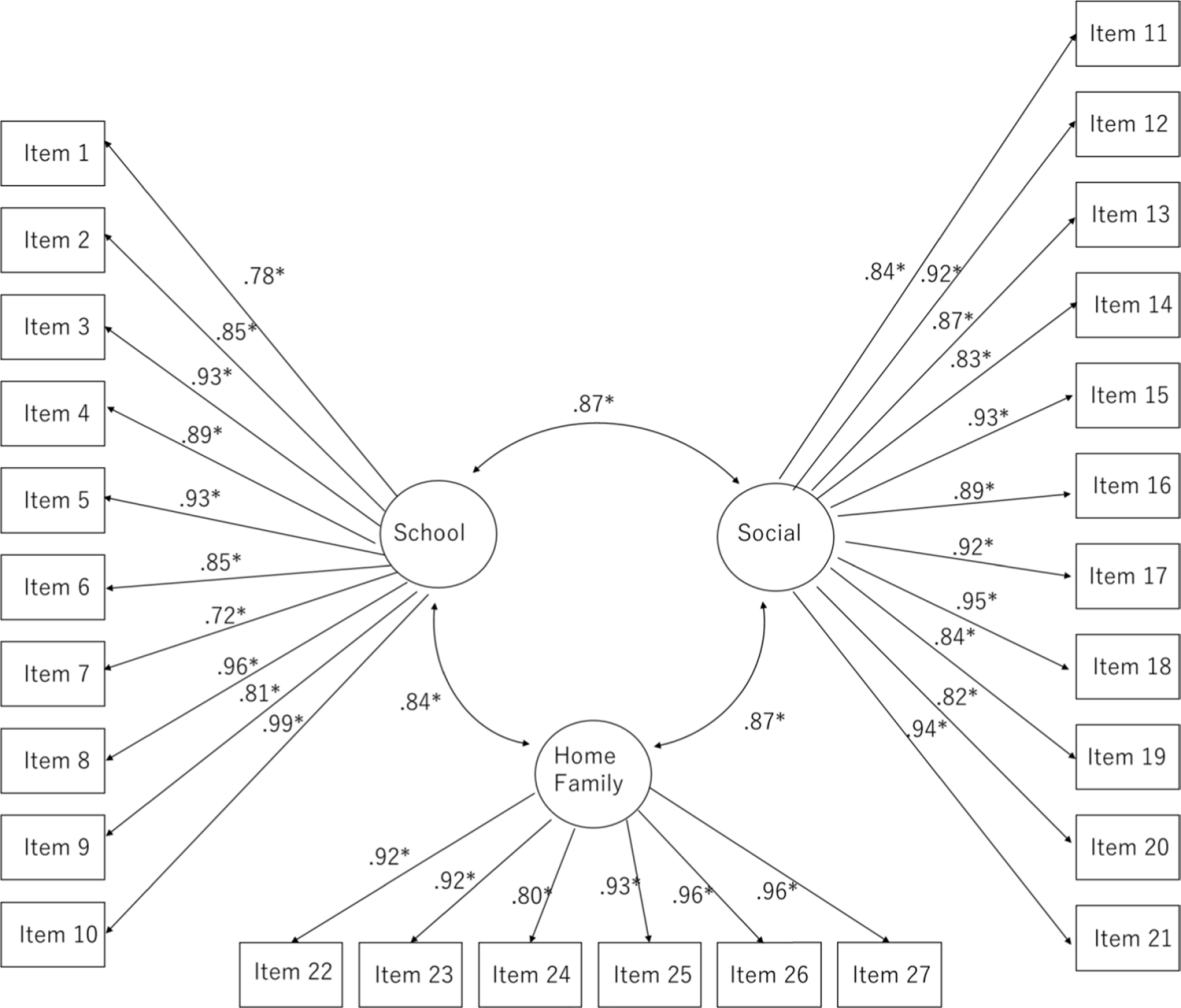
Three-factor model of CAIS-P

CFA was conducted to examine whether the factor structure of the CAIS-P to Japanese samples is the same as the factor structure to Western samples. The three-factor model is illustrated in Fig. 1. The three-factor model yielded fit indices of CFI = 0.966, RMSEA = 0.062, and SRMR = 0.055. The factor loadings ranged from 0.72 to 0.99 for school activity, 0.82 to 0.95 for social activity, and 0.80 to 0.96 for home/family activity.

### Convergent and Discriminant Validity of CAIS-P

We estimated Spearman’s rank correlation coefficient to test the convergent and discriminant validity of the CAIS-P. The correlation coefficients between CAIS-P, SCAS-P, and CDI were calculated (Table 3). The total and factor scores of the CAIS-P were moderately to strongly correlated with the SCAS-P (*r* = 0.44 to 0.52) and the CDI (*r* = 0.41 to 0.51). There was no significant difference in the correlation coefficient between CAIS-P/SCAS-P and CAIS-P/CDI (*t* (397) = 0.10, *p* = 0.913). There was no significant difference in the correlation coefficient between any factor score of CAIS-P/SCAS-P and CAIS-P/CDI (school activity: *t* (397) = -1.57, *p* = 0.116, social activity: *t* (397) = 1.80, *p* = 0.071, home/family activity: *t* (397) = 0.77, *p* = 0.442).

**Table 3:** Correlation coefficients of each measure

	CAIS-P	School	Social	Home/Family	SCAS-P
School	0.89*				
Social	0.78*	0.52*			
Home/Family	0.70*	0.53*	0.54*		
SCAS-P	0.52*	0.44*	0.50*	0.45*	
CDI	0.51*	0.51*	0.41*	0.41*	0.39*

CAIS-P = Child Anxiety Impact Scale-Parent version, School = School activity, Social = Social activity, Home/Family = Home/Family activity, SCAS-P = Spence Child Anxiety Scale-Parent version, CDI = Child Depression Inventory

*p < 0.001

The partial correlation coefficients between CAIS-P and SCAS-P were calculated (Table 3).

We controlled for the effect of depressive symptoms on the partial correlation coefficients. The total CAIS-P score was moderately correlated with SCAS-P, even though the effect of CDI was controlled (partial *r* = 0.40, *p* < 0.001). The partial correlation coefficients for each factor of CAIS-P and SCAS-P were also significant after controlling for the effect of CDI (School activity: partial *r* = 0.30, *p* < 0.001; Social activity: partial *r* = 0.40, *p* < 0.001; Home/family activity: partial *r* = 0.34, *p* < 0.001).

### Reliability and Information Function of CAIS-P

The unidimensionality of each factor in CAIS-P was confirmed using principal component analysis before we conducted the IRT. Since the first component explained more than 20% of the variance for all the factors (school activity = 60%, social activity = 60%, home/family activity = 69%), the unidimensionality of each factor was confirmed (Nguyen et al., 2014).

We estimated the discrimination and difficulty parameters for school activity, social activity, and home/family activity factors (Table 4).

**Table 4:** Discrimination and difficulty parameters for each item

	Discrimination	Difficulty		
		> = 1	> = 2	> = 3
School				
1. Getting to school on time in the morning	1.14	1.43	2.25	2.55
2. Giving oral reports or reading out loud	1.61	1.02	2.03	2.73
3. Writing in class	2.63	1.14	1.87	2.48
4. Taking tests or exams	2.24	0.91	1.70	2.54
5. Completing assignments in class	2.96	0.94	1.84	2.36
6. Doing homework	1.83	0.53	1.64	2.55
7. Getting good grades	1.25	0.26	1.41	2.41
8. Doing fun things during recess or free time	1.95	1.21	1.96	2.89
9. Concentrating on his/her work	1.62	0.11	1.33	2.20
10. Eating lunch with other kids	2.16	1.47	2.11	3.09
Social				
11. Making new friends	1.33	0.90	1.94	2.90
12. Leaving the house	2.32	1.16	1.84	2.53
13. Talking on the phone	1.73	1.10	1.92	2.68
14. Being with a group of strangers	1.59	0.58	1.69	2.36
15. Going to a friend’s house during the day	2.70	1.01	1.80	2.31
16. Spending the night at a friend’s house	2.16	0.88	1.78	2.32
17. Going to a sports event or ball game	2.42	1.16	1.96	2.51
18. Going shopping or trying on clothes	3.06	1.17	1.87	2.55
19. Going on a date	1.51	1.50	2.21	2.60
20. Having a boyfriend/girlfriend	1.34	1.46	2.22	2.70
21. Eating in public	2.62	1.35	2.09	2.60
Home/Family				
22. Getting ready for bed at night	2.59	0.96	1.76	2.25
23. Sleeping at night	2.73	1.04	1.83	2.38
24. Getting along with his/her brothers or sisters	1.46	1.08	1.81	2.55
25. Getting along with his/her parents	2.20	1.10	1.86	2.55
26. Visiting relatives	3.00	1.21	1.98	2.71
27. Having relatives visit	2.63	1.24	2.05	2.60

**Fig. 2:**
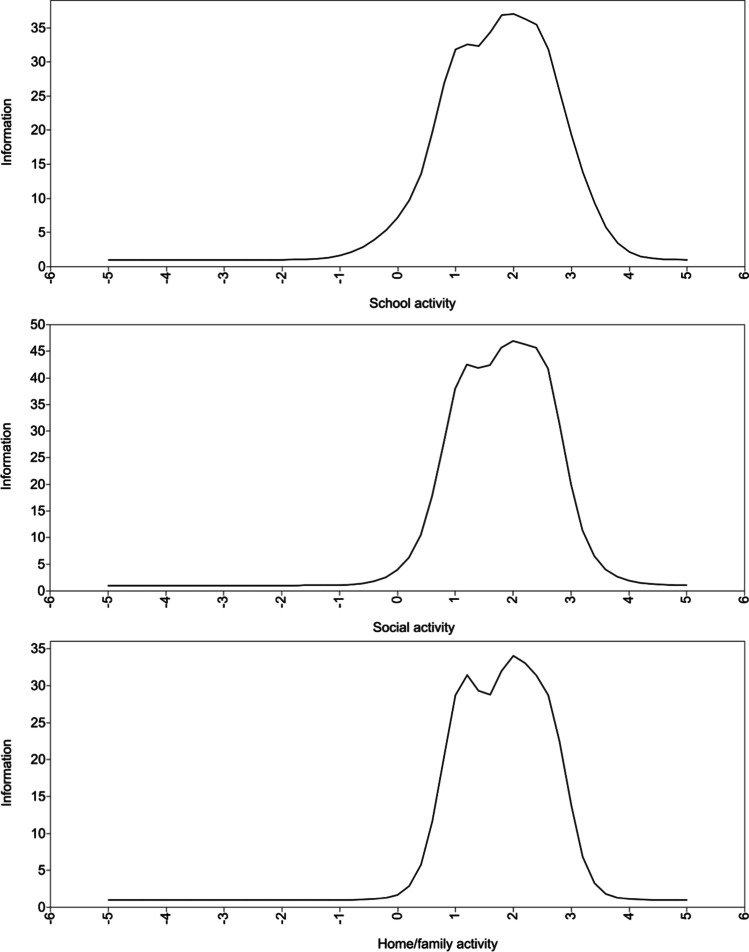
Test information function curve

The discrimination parameters ranged from 1.14 to 2.96, and the difficulty parameters ranged from 0.11 to 3.09 for the school activity factor. For the social activity factor, the discrimination parameters ranged from 1.33 to 3.06, and the difficulty parameters ranged from 0.88 to 2.90. In the home/family activity factor, the discrimination parameters ranged from 1.46 to 3.00, and the difficulty parameters ranged from 0.96 to 2.71. Based on the discrimination and difficulty parameters of each factor, we developed a test information function curve to show the characteristic and amount of information in each factor (Fig. 2).

## Discussion

The purpose of the present study was to develop the Japanese version of the CAIS-P and examine its reliability and validity in a community sample. We conducted a confirmatory factor analysis to confirm the factorial validity of the Japanese version of the CAIS-P, and convergent and discriminant validity were examined using Spearman’s rank correlation coefficient. We also conducted IRT to determine the reliability and test information of the Japanese version of the CAIS-P. This is the first study that conducted IRT to examine the characteristics of CAIS-P.

As hypothesized, the Japanese version of the CAIS-P consists of the same factor structure as the original version. We developed a three-factor model based on the original CAIS-P (Langley et al., 2004). Considering that model fit indexes of three factor models were above the cut-off values and all factor loadings exceeded 0.7 (Hair et al., 2011), the three-factor structure consisting of school activity, social activity, and home/family activity factors was supported in the Japanese version of CAIS-P.

The second hypothesis, which stated that we would find a moderate correlation between the Japanese version of the CAIS-P and the SCAS-P, was confirmed. This result was consistent with a prior study that found a moderate correlation between the total and factor scores of the CAIS-P and anxiety symptoms measured by the Screen for Child Anxiety Related Emotional Disorders scale (*r* = 0.47 to 0.63; Langley et al., 2014). Higher levels of anxiety symptoms have a greater impact on the daily life of children, causing difficulty in school, social, and home/family activities. Therefore, the convergent validity of the Japanese version of the CAIS-P was confirmed in this study.

The third hypothesis, which stated that we expected to find a stronger correlation between the CAIS-P and the SCAS-P than between the CAIS-P and the CDI, was not confirmed. The correlation between CAIS-P and the CDI in the present study was similar to a previous study (*r* = 0.47, *p* < 0.001; Langley et al., 2004), but we are not aware of any previous studies that have compared the correlation between the CAIS-P/SCAS-P and the CAIS-P/CDI. Our result may be explained by the fact that anxiety and depressive symptoms often co-occur and both can negatively impact children’s daily lives (Garber & Weersing, 2010). A moderate correlation between the CAIS-P and the CDI in this study does suggest that depressive symptoms also have an impact on children’ s daily lives. However, the CAIS-P was still significantly correlated to SCAS-P, even when the effect of depressive symptoms was considered. This indicates that although the CAIS-P responses were partially related to depressive symptoms, it measures the impact of anxiety on children’s lives.

The results of the IRT analysis indicate that the Japanese version of the CAIS-P has high reliability and information for measuring the impact of children’s anxiety on daily life. The discrimination and difficulty parameters suggested that each factor of the CAIS-P is a highly reliable measure. No previous studies have conducted an IRT analysis on CAIS-P. The benefit of using IRT is that it can identify the characteristics of the items and scale, the results of which can be generalized to different groups. In the present study, all the items exceeded 0.65 on the discrimination parameter, indicating that the items provide at least moderate information and are appropriate for measuring the impact of a child’s anxiety on daily life (Baker, 2001). All the items in the home/family activity factor are informative for children with high traits; however, some items on the school and social activity factors are also informative for children with moderate traits. In relation to school activity, the difficulty parameter of =  > 1 for items 4, 5, 6, 7, and 9 was relatively small compared to other items. These items are all related to school grades, and this suggests that children who do not experience marked anxiety problems are also likely to have at least some problems in situations related to academic performance. The difficulty parameter of =  > 1 for items about interaction with new people (items 11, 14, and 16) was relatively small compared to other items on the social activity factor, indicating that interacting with strangers can often be difficult for children, even those who have few general problems related to anxiety. The test information function curve indicated that each factor had high information for individuals with high trait levels. All factors have low informativity below 0 and gain informativity as the individuals’ trait level increases, indicating that the CAIS-P is a highly informative measure for individuals with high trait levels of anxiety problems in Japan. However, whether this result can be replicated in other cultures is yet to be ascertained. Therefore, future research examining how much information each item holds in Western cultures is warranted, to clarify the cultural differences in the impact of anxiety on each activity.

Our findings provide preliminary evidence for the suitability of the use of the CAIS-P in Japan and its potential for future application in clinical settings. They show that the Japanese version of the CAIS-P is suitable for children with high trait levels of anxiety problems and therefore warrants further evaluation in clinical populations. In Japan, there are currently only a few options for assessing clinical outcomes in intervention studies for childhood anxiety; the Japanese CAIS-P provides a quick and easy means to assess functional impairment associated with a child’s anxiety symptoms, as well as an opportunity to explore potential differences in child anxiety impairment across Eastern and Western countries. Previous research indicates that the CAIS-P can accurately identify recovery from child anxiety disorders in the UK population (Evans et al., 2016), and it will be important for future studies to examine the capacity of the Japanese version to detect recovery from anxiety disorders.

### Limitations

There were several limitations in the current study. This study was conducted on a community sample; hence, it will be important for future studies to evaluate the measure in clinical settings and among children who meet the diagnostic criteria for an anxiety disorder. It is also important to acknowledge that we were unable to collect information on participants’ socioeconomic background which will be important to consider in future studies. We also only set out to develop and evaluate a parent-report version of the CAIS-P. Given the encouraging findings from this study, it will be important for future studies to consider developing a child-report Japanese CAIS. Further, the convergent and discriminant validity analyses were limited to the SCAS-P and CDI. There are several measures (e.g., Child Behavior Checklist and The Children’s Global Assessment Scale) that can be used to examine the validity of the CAIS-P. Therefore, further examination using other measures is warranted to provide more comprehensive evidence of the validity of the CAIS-P. Additionally, test–retest reliability was not examined in this study and should be assessed in future evaluations. Finally, this study was conducted during the pandemic of COVID-19, which may have had an impact on children’s anxiety, daily life, and parents’ responses to the CAIS-P.

## Conclusion

Even with the aforementioned limitations, this study contributes to the field of childhood anxiety. We developed the Japanese version of the CAIS-P and provided initial evidence to support its validity and reliability. This study revealed that the Japanese version of the CAIS-P is an informative measurement for children with high trait anxiety and has potential for future application in clinical trials in Japan.

## Data Availability

The datasets generated and/or analyzed during the current study are available from the corresponding author on reasonable request.
